# Visualisation of newly synthesised collagen *in vitro* and *in vivo*

**DOI:** 10.1038/srep18780

**Published:** 2016-01-07

**Authors:** Corien Oostendorp, Peter J.E. Uijtdewilligen, Elly M. Versteeg, Theo G. Hafmans, Ellen H. van den Bogaard, Paul K.J.D. de Jonge, Ali Pirayesh, Johannes W. Von den Hoff, Ernst Reichmann, Willeke F. Daamen, Toin H. van Kuppevelt

**Affiliations:** 1 Radboud university medical center, Radboud Institute for Molecular Life Sciences, Department of Biochemistry, Nijmegen, The Netherlands; 2 Radboud university medical center, Radboud Institute for Molecular Life Sciences, Department of Dermatology, The Netherlands; 3 Radboud university medical center, Radboud Institute for Molecular Life Sciences, Department of Urology, Nijmegen, The Netherlands; 4 Ghent University Hospital, Burn Center, Department of Plastic and Reconstructive Surgery, Ghent, Belgium.; 5 Radboud university medical center, Department of Orthodontics and Craniofacial Biology, Nijmegen, The Netherlands; 6 University Children’s Hospital Zurich, Department of Surgery, Tissue Biology Research Unit, Zurich, Switzerland

## Abstract

Identifying collagen produced *de novo* by cells in a background of purified collagenous biomaterials poses a major problem in for example the evaluation of tissue-engineered constructs and cell biological studies to tumor dissemination. We have developed a universal strategy to detect and localize newly deposited collagen based on its inherent association with dermatan sulfate. The method is applicable irrespective of host species and collagen source.

Collagen, the most abundant protein family in the human body, plays a pivotal role in the organization of tissues and organs, and is a major determinant during organogenesis. In the field of tissue engineering and regenerative medicine, type I collagen is a key biomaterial^1^ whereas in other fields, notably cancer research, collagen gels are frequently used in 3D studies to the migrational behavior of cells^2^.

A common challenge in the field is to make a distinction between the collagen synthesized by cells and the (abundant) pre-existing collagen present in the biomaterial. Antibodies raised against collagens are of limited use due to the highly conserved nature of collagens^3^ and the associated cross reactivity between collagen from different species. Other methods like metabolic radiolabeling and mass spectrometry^4^ are laborious and do not provide information about the topography and organization of the newly synthesized collagen fibers.

In this study we evaluated newly synthesized fibrillar collagen (*e.g.* type I collagen), by making use of the inherent and intrinsic association of the glycosaminoglycan dermatan sulfate with collagen fibrils. Dermatan sulfate is the glycosaminoglycan part of the proteoglycans decorin and biglycan, which are both collagen fibril-associated molecules that play a role in the regulation of collagen fibril diameter. These proteoglycans remain present on the mature collagen fibril (Fig. 1a, cartoon), and therefore dermatan sulfate is associated with collagen fibrils^5^,^6^. The technique described here is based on the selective detection of dermatan sulfate using the single chain variable fragment antibody GD3A12^7^, combined with the absence of dermatan sulfate in experimentally or commercially produced biomaterials. We tested the technique both *in vivo* and *in vitro* using a number of collagenous biomaterials including gels cultured with human fibroblasts with or without keratinocytes (denovoSkin and denovoDerm respectively)^8^, experimental and commercially available scaffolds, and glycerol preserved acellular human dermis (Glyaderm^®^)^9^.

## Results

**Figure 1:**
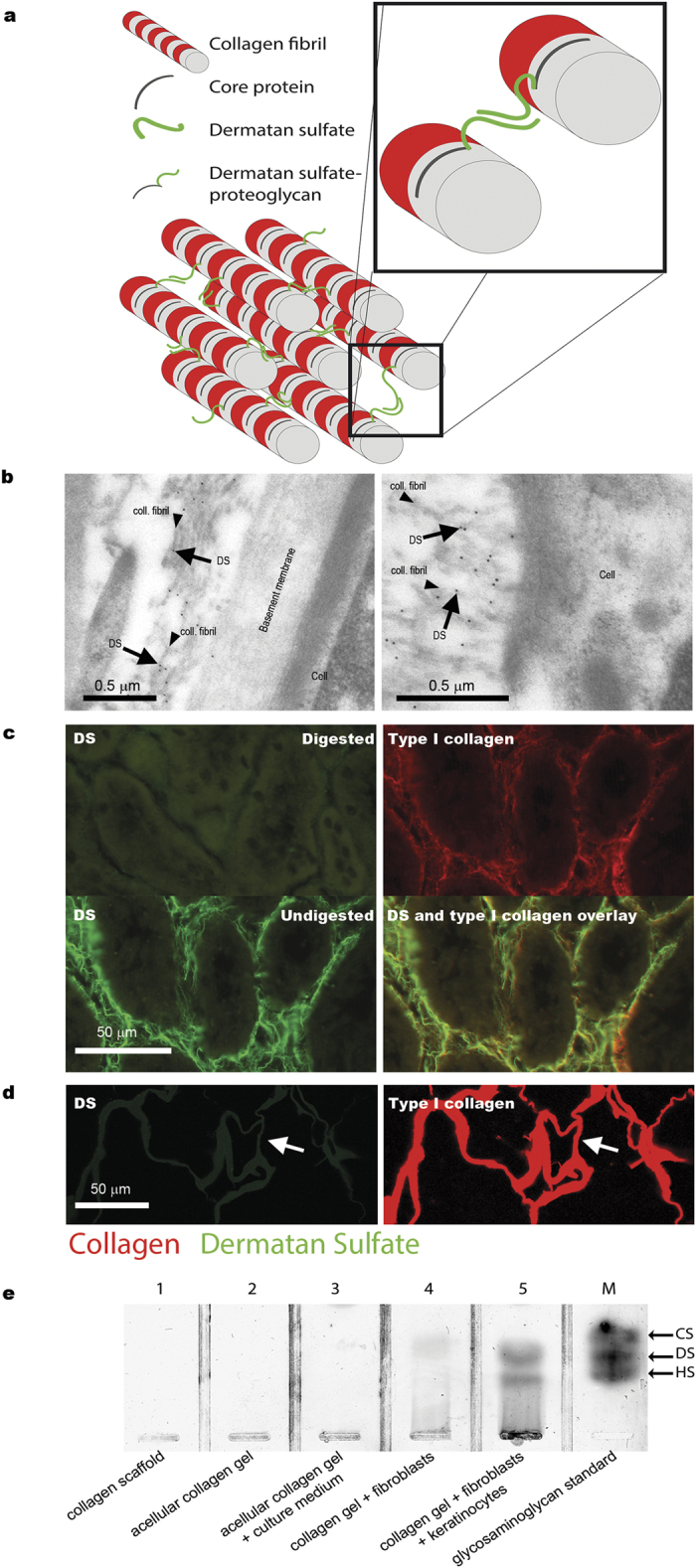
Overview and validation of strategy to identify newly synthesized collagen by dermatan sulfate. **(a)** Cartoon illustrating the intrinsic association of dermatan sulfate with collagen fibrils. **(b)** Identification of collagen fibrils using the anti-dermatan sulfate single chain antibody GD3A12. Arrows indicate immunogold labeling on collagen fibrils (rat kidney tissue, Bowman’s capsule), but not on other structures such as cells and basement membranes. **(c)** Specificity of the anti-dermatan sulfate antibody as evidenced by loss of immunostaining after digestion of dermatan sulfate by chondroitinase B (rat kidney tissue). Note co-localization of dermatan sulfate and type I collagen. (**d,e**) Absence of dermatan sulfate in pre-seeded/pre-implanted collagenous biomaterials as indicated by (**d**) immunostaining for dermatan sulfate (antibody GD3A12), (**e**) biochemical analysis of dermatan sulfate (agarose gel electrophoresis). In (d) arrows indicate identical areas stained for dermatan sulfate and type I collagen. In (**e**), lanes 1–3 represent acellular collagen gels/scaffolds, whereas lanes 4 and 5 represent cellularized gels. M, marker containing 5 ng each of chondroitin sulfate (CS), dermatan sulfate (DS) and heparan sulfate (HS). coll.fibril: collagen fibril.

To evaluate the potential of the anti-dermatan sulfate antibody to identify collagen fibrils we applied immuno-electron microscopy using rat kidney cryosections. Antibody reactivity, as visualized by gold sphere-labeled protein A, was confined to collagen fibrils whereas other structures like cells and basement membranes did not stain (Fig. 1b). Using immunofluorescence, antibody staining for dermatan sulfate was shown to co-localize with type I collagen, and was abolished by pretreatment of the sections with chondroitinase B, which specifically digests dermatan sulfate (Fig. 1c).

### Absence of dermatan sulfate in the biomaterials

All collagenous biomaterials used were tested for the presence of dermatan sulfate, using immunohistochemical and/or biochemical techniques. Using immunofluorescence, dermatan sulfate could not be detected in any of the biomaterials (Fig. 1d, and supplementary Figure S1). In addition, using a highly sensitive silver staining method, dermatan sulfate could not be observed in collagen scaffolds (Fig. 1e, lane 1) or in collagen gels (Fig. 1e, lane 2 and 3).

### Collagen deposition *in vitro* and *in vivo*

**Figure 2:**
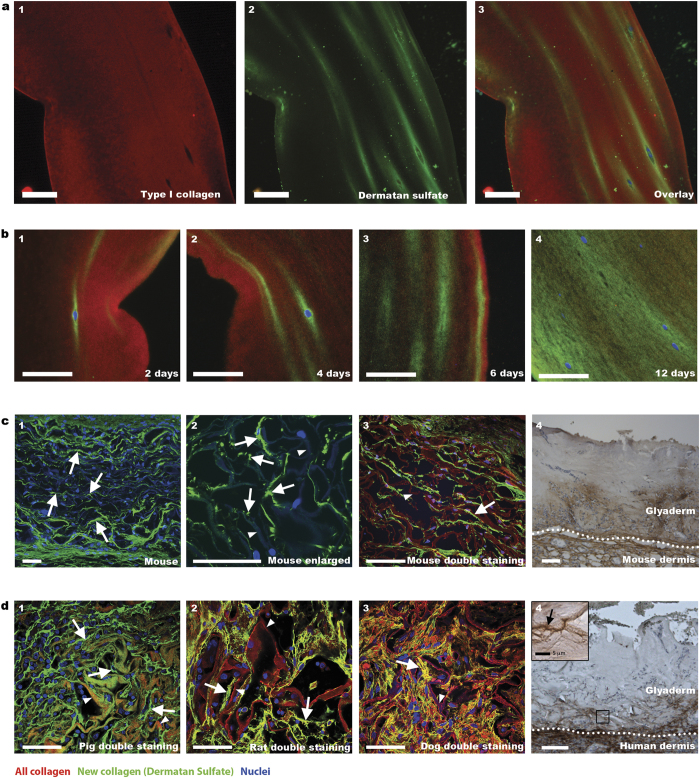
Detection of newly synthesized collagen fibrils in cellularized/implanted collagenous biomaterials. **(a)** Collagen gel cultured for 6 days with human fibroblasts. Newly deposited collagen is indicated by green dermatan sulfate staining (a2,a3), whereas all collagen is indicated by red type I collagen staining (a1,a3). **(b)** Location of newly deposited collagen in collagen gels cultured in time with fibroblasts/keratinocytes. Note increase of new collagen over time (b1-4). **(c)** Newly deposited collagen fibrils (arrows) in a collagen scaffold (arrowhead), two weeks after subcutaneous implantation in mice (c1-3) (for clarity, autofluorescence of background collagen was enhanced). For Glyaderm^®^ (acellular human dermis), new collagen is indicated by brown staining (c4). **(d)** Newly deposited collagen fibrils (arrow) in various species (d1-4) after implantation of a collagen scaffold (arrowhead) in pig (1 month) (d1), Integra^®^ (arrow head) in rat (1 week) (d2), Integra^®^ (arrow head) in dog (4 weeks) (d3), and Glyaderm^®^ in human (inset shows fibrillar structure) (d4). Scale bars are 50 μm unless indicated otherwise.

Having demonstrated the capacity of the antibody to detect collagen fibrils by virtue of its association with dermatan sulfate, and having established the absence of dermatan sulfate in collagenous biomaterials, we studied newly synthesized collagen fibrils produced by cells both *in vitro* and *in vivo*, using dermatan sulfate staining. Fibroblasts cultured *in vitro* in a collagenous gel produced collagen as evidenced by the presence of dermatan sulfate, which co-localized with type I collagen. Use of anti-type I collagen antibody did not discriminate between bovine collagen from the scaffold and the human collagen produced by the fibroblasts (Fig. 2a1–3). Dermatan sulfate staining, however, indicated the location of newly synthesized human collagen and was not present in the bovine scaffold collagen. Dermatan sulfate was also identified biochemically, and was detected only in cellularized collagen gels, and not in gels without cells (Fig. 1e, lane 4). The location of newly synthesized collagen was time dependent, and initially present only at the perimeter of the fibroblasts (Fig. 2b1). At later stages (*e.g.* 12 days of culturing) collagen was also located further away from the cells, and eventually most of the original gel contained newly synthesized collagen (Fig. 2b4). These results were confirmed biochemically, showing increased amounts of dermatan sulfate as a function of time (Fig. 1e).

Glycosaminoglycans are evolutionary highly conserved structures that are found throughout vertebrates as well as invertebrates^10^. It may therefore be expected that the anti-dermatan sulfate antibody can be used irrespective of the species that deposits the collagen. To evaluate this we stained collagen scaffolds implanted in different animal species and in humans. The following samples were used: 1) a flat collagen scaffold implanted subcutaneously in mice, 2) a tubular collagen scaffold implanted in the ureter of pigs, 3) a commercial collagen-chondroitin sulfate skin substitute (Integra^®^) implanted in a full-thickness skin defect in rats^11^, 4) Integra^®^ implanted in a soft tissue palatal defect in dogs^12^, 5) Glyaderm^®^ (acellular human dermis) implanted in a full-thickness skin defect in mice^13^ and 6) Glyaderm^®^ clinically applied in full-thickness skin defects in humans^14^. We were able to visualize the dermatan sulfate (and hence newly deposited collagen) in all species tested, indicating the robustness and species independency of the procedure (Fig. 2c,d). In line with the ingrowth of cells from the surrounding tissue into the scaffold, newly deposited collagen fibers were most prominent at the border of the scaffold, whereas deeper in the scaffold they were thinner and less abundant (Fig. 2c1). Small collagen deposits could easily be identified (Fig. 2c2). The newly formed collagen was generally oriented in the same direction as the fibers from the original scaffold (*e.g.* in a parallel orientation, see Fig. 2c3). In the pig model (Fig. 2d1), newly formed collagen was clearly present alongside the collagen fibers of the original implanted tubular collagen scaffold. Newly formed collagen fibers could also be easily identified in rats and dogs after implantation (7 and 28 days respectively) with Integra^®^ (Fig. 2d2,d3). Integra^®^ itself was not stained by the anti-dermatan sulfate antibody (supplementary Figure S1), even though the closely related glycosaminoglycan chondroitin sulfate is abundantly present in this commercially available skin substitute. The method was also applicable using the human skin derived Glyaderm^®^. Full-thickness wounds in mice treated with Glyaderm^®^^13^ showed deposition of new collagen 8 days after implantation (Fig. 2c4). Please note that due to the strong autofluorescence of elastic fibers in Glyaderm^®^ we used bright field instead of fluorescence microscopy. Finally, the method was probed in a clinical setting in which burn patients were treated using Glyaderm^®^^14^. Biopsies taken 7 days after implantation clearly show new collagen in the dense collagenous environment of Glyaderm^®^ at the border of the wound bed (Fig. 2d4). Collagen fibers of the surrounding native tissue (*i.e.* in tissue not formed within the biomaterial) were also positive for dermatan sulfate in all species tested, as expected.

## Discussion

The results presented above indicate that the anti-dermatan sulfate antibody GD3A12 is suitable to species independently detect newly formed collagen. Previously, analysis has been hampered by the inability to (immuno)histologically distinguish newly formed collagen from biomaterial/scaffold collagen. The technique described here offers a solution to this problem. However, it is not without potential pitfalls. Although the vast majority of dermatan sulfate is associated with collagen as part of the proteoglycans decorin and biglycan^5^, a small fraction may be present associated with other structures such as elastin and fibrillin-containing microfibrils^15^. In addition, dermatan sulfate may be part of the proteoglycan versican^16^ associated with elastic fibers^17^. However, in this study we did not observe any association of dermatan sulfate with elastic fibers detected either by autofluorescence or by anti-elastin antibodies (see double staining with dermatan sulfate, supplementary Figure S2), indicating that such an association was not present in the tissues studied here.

Next to the obvious use in regenerative medicine, the proposed method may be applied to other fields of research including cancer biology. Tumor cells spread and invade into the surrounding tissues while remodeling the extracellular matrix. It has been suggested that in doing so tumor cells make use of newly deposited collagen fibrils^18^. A widely used 3D model to study the migrational behavior of tumor cells is the use of collagen gels. The technique described here may be of value in further defining the role of newly formed collagen fibrils in tumor biology using such models^2^.

In conclusion, the detection of newly synthesized collagen based on its association with dermatan sulfate and applying the single chain antibody GD3A12 represents an inexpensive, fast and easy technique to evaluate the presence and orientation of *de novo* synthesized collagen fibrils in collagen based biomaterials. As such it can be applied in many research areas including tissue engineering and tumor biology.

## Materials and methods

### Materials

Papain, barium acetate, paraformaldehyde, 3,3′-diaminobenzidine tetrahydrochloride (DAB), 4′,6-diamidino-2-phenylindole dihydrochloride (DAPI) and protein A were from Sigma, St. Louis MO, USA. Chondroitinase B was from IBEX, Montreal, Quebec, Canada. Agarose and gel bond film were from Lonza, Rockland, USA, and Tissue Tek from Sakura Finetek Europe BV, Alphen aan den Rijn, The Netherlands. Tris-HCl was from Invitrogen, Carlsbad, CA, USA. Sodium chloride and magnesium acetate were from Merck, Darmstadt, Germany. Lowicryl HM20 was from Aurion, Wageningen, The Netherlands and Mowiol 4-88 mounting medium was from Calbiochem, San Diego, CA, USA. Bovine serum albumin (BSA) was from PAA laboratories, Pasching, Austria. The following antibodies were used: rabbit anti-bovine type I collagen IgG from Millipore, Cambridge, UK; mouse anti-bovine elastin IgG from Sigma; rabbit anti-VSV IgG from Rockland, Gilbertsville, PA, USA; mouse anti-VSV IgG from mouse hybridoma cell line P5D4 from the American Type Culture Collection, Rockville, MD, USA; peroxidase labeled mouse anti-VSV IgG from Sigma and mouse anti-Penta-His IgG from QIAGEN GmbH, Hilden, Germany; goat anti-mouse IgG Alexa Fluor 488 conjugated and goat anti-rabbit IgG Alexa Fluor 594 conjugated from Invitrogen, Eugene, OR, USA. The single chain variable fragment antibody GD3A12 selective for dermatan sulfate was obtained as described^7^,^19^. As a source of this antibody, periplasmic fractions isolated from bacteria expressing the antibody were used^20^. The antibody contains a VSV and a HIS tag.

### Histology

Paraffin-embedded and frozen tissues were sectioned at 5 μm thickness. Paraffin sections were deparaffinized in xylene for 3 × 5 min, followed by a descending series of ethanol and processed for immunohistochemistry. Cryosections were air-dried before staining.

#### Immunofluorescence stainings

To stain for dermatan sulfate, deparaffinized sections were blocked for 15 min with 1% BSA in Tris buffered saline (TBS, 50 mM Tris-HCl pH 7.0 containing 150 mM NaCl). All further incubations were performed at ambient temperatures for 45 min and sections were washed 3 × 5 min with TBS in between incubations. Antibodies were diluted in 1% BSA in TBS. Paraffin sections were incubated with antibody GD3A12 (1:5 - 1:20), followed by incubation with mouse anti-VSV antibody P5D4 (1:10) and an Alexa Fluor 488-conjugated goat anti mouse antibody. For double staining with type I collagen, antigen retrieval using citrate buffer was applied. Paraffin sections were pretreated with citrate (10 mM sodium citrate, pH 6.0) for 20 min at either 95 °C (tissues) or ambient temperature (cultured gels). In one occasion boiling temperature was used, but this caused damage to the sections. Sections were extensively washed with TBS to remove the citric acid buffer and blocked with TBS/BSA. Anti-type I collagen antibody (1:500–1:1500) was applied and visualized using an Alexa Fluor 594-conjugated goat anti-rabbit antibody. For detection of dermatan sulfate in implanted collagen scaffolds in mice, a rabbit anti-VSV antibody (1:500) was used and visualized using goat anti-rabbit IgG Alexa Fluor 488. In the case of double staining of dermatan sulfate and type I collagen in mice, dermatan sulfate was visualized using Alexa Fluor 488 conjugated mouse anti-Penta-His antibody.

For double staining of sheep skin cryosections for elastin and dermatan sulfate, the sections were air-dried for 30 min. Hereafter, the procedure as described above was applied. Mouse anti-bovine elastin antibody was diluted 1:200 and visualized using goat anti-mouse IgG Alexa Fluor 594. Dermatan sulfate was visualized using rabbit anti-VSV IgG and goat anti-rabbit IgG Alexa Fluor 488.

For visualization of the nuclei, sections were incubated for 15 min with DAPI (10 μg/ml in PBS). After extensive washings with PBS, the sections were enclosed with Mowiol mounting medium.

For detection of dermatan sulfate in the human skin substitute Glyaderm^®^, containing autofluorescent elastic fibers, peroxidase conjugated mouse anti-VSV IgG (1:100) and DAB were used.

Omission of the antibody GD3A12 was taken as a control, and was negative in all cases.

#### Digestion of dermatan sulfate to evaluate specificity of GD3A12

To evaluate the specificity of the antibody GD3A12 for dermatan sulfate, cryosections were digested overnight at 37 °C with 20 mU/ml chondroitinase B in 25 mM Tris-HCl pH 8.0 containing 2 mM magnesium acetate. The next day, the digestion was repeated with 20 mU/ml chondroitinase B for another 2 h. As a control, sections were incubated in buffer without enzyme.

#### Microscopic imaging, equipment and settings

Images of the *in vitro* cultured collagen gels were taken with a Leica DM6000 B microscope equipped with a Leica DFC 480 camera (20x objective), using Leica Application suite V4.3.0. The exposure time was kept constant for all measurements. DAPI, Alexa Fluor 488 and 594 were excited with a mercury HBO100 lamp using the excitation filters BP410/15 nm BP490/20 nm and BP562/40 nm respectively, and the emission was collected after filtering with 430, 500, 593 dichroic mirrors respectively.

All other images were captured with the Olympus FV1000 Confocal Laser Scanning Microscope. Photos were imaged using a 20× objective and a 60× objective. DAPI, Alexa Fluor 488 and Alexa Fluor 594 were excited at 405 nm, 488 nm and 559 nm, respectively. Using a combination of the beam splitters SMD490 and SDM560, the emission was collected with the emission filters BA430-470, BA505-540 and BA575-675 for DAPI, Alexa Fluor 488 and Alexa Fluor 594, respectively.

For Figs 1c and 2a,b and supplementary Figure S1a, the microscope settings were as follows: space resolution 2560 × 1920, pixel dimension 0.012 pixels/μm, image depth 32 (RGB), excitation filters: 410/15 (DAPI), 490/20 (AF488), 562/20 (AF594) and gamma correction was set at 1. For Figs 1d and 2c,d, the settings were as follows: space resolution 1024 × 1024, pixel dimension 1.61 pixels/μm, image depth 32 (RGB), excitation filters 405 (DAPI), 488 (AF488), 559 (AF594), emission filters BA430-470 (DAPI), BA505-540 (AF488), BA575-675 (AF594). Dichroic beam splitters 490 9DAPI) and 560 (AF488) were used. No gamma correction was applied.

Image processing was performed using ImageJ 1.48v (National Institutes of Health, USA). Before merging, both brightness and contrast were adjusted similarly for all photos including the controls.

#### Immuno-electron microscopy

To evaluate the reactivity of the antibody for dermatan sulfate on collagen fibrils, immuno-electron microscopy was performed on lowicryl HM20 embedded rat kidney samples^21^. The tissue was incubated for 3 h in Somogyi solution [0.1 M phosphate buffer (pH 7.3) containing 4% formaldehyde, 0.05% glutaraldehyde and 0.2% picric acid]^22^. After cutting, 200 μm sections were frozen in liquid propane at −190 °C. Using freeze-substitution (Leica-KF80) the sections were embedded in lowicryl HM20. Ultrathin sections were mounted on nickel grids. For immunostaining sections were blocked with 0.25% BSA in phosphate buffered saline (BSA/PBS), followed by an overnight incubation at 4 °C with antibody GD3A12 (5× diluted periplasmic fraction in BSA/PBS). After washing, bound GD3A12 was visualized using 10 nm gold-sphere labeled protein A (1:400 in BSA/PBS) prepared according to Slot *et al.*^23^. Subsequently, sections were washed in PBS, post-fixed for 5 min in 2.5% glutaraldehyde in 0.1 M phosphate buffer (pH 7.4), washed with distilled water, and post-stained with uranyl acetate. Sections were examined using a JEOL 1010 electron microscope.

### Agarose gel electrophoresis

To analyze the presence of glycosaminoglycans including dermatan sulfate in collagen scaffolds/gels, agarose gel electrophoresis was performed. To 40 mg dry weight of the samples, 2.5 U/ml papain was added to a total volume of 500 μl in order to digest proteins. 0.5 μl of the samples was loaded on a 1 mm thick 1% agarose gel in 50 mM Ba(Ac)_2_, pH 5.0, casted on a gel bond film. A marker was included containing 5 ng of chondroitin sulfate (CS), 5 ng dermatan sulfate (DS) and 5 ng heparan sulfate. The gel was run at 30 mA in electrophoresis buffer (50 mM Ba(Ac)_2_, pH 5.0) until the front of the loading dye had moved about 8 cm into the agarose gel. Subsequently, the agarose gel was stained with silver as described by Van de Lest *et al.*^24^.

### Gels and scaffolds

All experimental protocols described by Sun *et al.*^25^, Nillesen *et al.*^11^, van Kilsdonk *et al.*^13^, de Jonge *et al.*^26^, and Ophof *et al.*^12^ were approved by the Institutional Animal Welfare Committee (DEC) of the Radboud university medical center, Nijmegen, The Netherlands. The experimental procedures described by Braziulis *et al.*^8^ were approved by the Ethics Committee of the Canton Zürich (KEK), Switzerland. The study protocol as described by Pirayesh *et al.*^14^ was approved by the Ghent University Hospital Ethics Committee.

All experiments were carried out in accordance with the guidelines of the Institute of Laboratory Animal Research^27^ and the declaration of Helsinki principles.

#### Cellularized collagen gels

The dermal (denovoDerm) and dermal-epidermal (denovoSkin) collagen gels were prepared using bovine type I telocollagen seeded either with primary fibroblasts only (denovoDerm), or with a combination of primary fibroblasts and keratinocytes (denovoSkin), isolated from skin biopsies from 3 individuals who had given informed consent, as described by Braziulis *et al.*^8^. 50,000 fibroblasts were cultured in DMEM supplemented with 10% fetal bovine serum (FBS), 10 mM 4-(2-hydroxyethyl)-1-piperazineethanesulfonic acid buffer (HEPES), 90 μg/ml streptomycin and 90 U/ml penicillin (all compounds from Invitrogen, Basel, Switzerland) for 2, 4 or 6 days in compressed collagen gels at 37 °C and 5% CO_2_. For the dermal-epidermal skin substitute, 500,000 keratinocytes were seeded on top of the dermal substitute and cultured for another 6 days (12 days in total) in serum free keratinocyte medium. Samples were fixed in 4% paraformaldehyde and embedded in paraffin.

#### Acellular collagen scaffolds implanted in mice

Flat porous collagen scaffolds were prepared as described by Sun *et al.*^25^ from a 0.4% (w/v) type I collagen suspension in 0.25 M acetic acid. After homogenization, the suspension was pipetted in a polystyrene mold, frozen and lyophilized resulting in porous collagen scaffolds. Hereafter, the collagen scaffolds were pre-incubated in 50 mM 2-(N-morpholino)ethanesulphonic acid pH 5.0 (MES) and crosslinked using 33 mM 1-ethyl-3-(3-dimethylaminopropyl) carbodiimide (EDC) and 6 mM N-hydroxysuccinimide (NHS) in 50 mM MES buffer containing 40% ethanol for 4 h. After multiple washing steps with subsequently 0.1 M Na_2_HPO_4_, 1 M NaCl, 2 M NaCl, and water, the scaffolds were frozen at −20 °C and lyophilized, followed by sterilization by γ-irradiation (25 kGy, Synergy Health, the Netherlands). Collagen scaffolds were incubated in sterile 0.9% NaCl and subcutaneously implanted in 7-weeks-old Balb/cByj mice. After two weeks, mice were sacrificed and tissue was dissected around the site of implantation, fixed in 4% paraformaldehyde and embedded in paraffin.

#### Tubular acellular collagen scaffolds implanted in pigs

Tubular collagen constructs of 6 cm in length and an inner diameter of 6 mm were prepared as described using a 0.5% (w/v) collagen suspension in 0.25 M acetic acid^28^. After lyophilization the tubes were crosslinked followed by extensive washing, as described above. Tubular scaffolds were kept in 70% ethanol before γ-sterilization. Sterilized tubular collagen scaffolds were implanted in the ureter of 4-months-old pigs, dissected after one month, fixed in 4% paraformaldehyde and embedded in paraffin^26^.

#### Integra^®^ implanted in rat and dog

In rats, Integra^®^, a commercially available collagen-chondroitin sulfate scaffold^29^, was implanted in a full thickness wound^11^. After 7 days, rats were sacrificed, the wound area dissected, fixed in 4% paraformaldehyde and embedded in paraffin^11^. In dogs, Integra^®^ was implanted according to the Von Langenbeck procedure for palatal repair^12^. Samples were taken 28 days post implantation. Tissue was fixed in 4% formaldehyde, decalcified in 20% formic acid and 5% sodium citrate^12^ and imbedded in paraffin.

#### Glyaderm^®^ implanted in mouse and human

Glyaderm^®^, a glycerol preserved acellular human dermis containing native collagen and elastic fibers^9^ was produced by the Euro Skin Bank, Beverwijk, The Netherlands. Full thickness wounds at the back of 8-week-old mice were implanted with Glyaderm^®^. After 8 days, mice were sacrificed, fixed in 4% paraformaldehyde and embedded in paraffin^13^.

Application of Glyaderm^®^ to a human wound bed was performed and described by Pirayesh *et al.*^14^. Informed consent was given by all patients. Full-thickness defects were engrafted with Glyaderm^®^. After 1 week, biopsies were fixed in 4% paraformaldehyde and embedded in paraffin.

## Additional Information

**How to cite this article**: Oostendorp, C. *et al.* Visualisation of newly synthesised collagen *in vitro* and *in vivo*. *Sci. Rep.* **6**, 18780; doi: 10.1038/srep18780 (2016).

## Supplementary Material

Supplementary Information
