# Supplementary material for: Visualisation of newly synthesised collagen in vitro and in vivo
Source: Sci Rep. 2016 Jan 7;6:18780. doi: 10.1038/srep18780 (PMC4704054; doi:10.1038/srep18780)
Supplement: Supplementary Information [file srep18780-s1.pdf]

Visualisation of newly synthesised collagen *in vitro* and *in vivo*

Corien Oostendorp[1,7], Peter J.E. Uijtdewilligen[1,7], Elly M. Versteeg[1], Theo G. Hafmans[1], Ellen H. van den Bogaard[2], Paul K.J.D. de Jonge[3], Ali Pirayesh[4], Johannes W. Von den Hoff[5], Ernst Reichmann[6], Willeke F. Daamen[1,8], Toin H. van Kuppevelt[1,8]*

Supplementary information

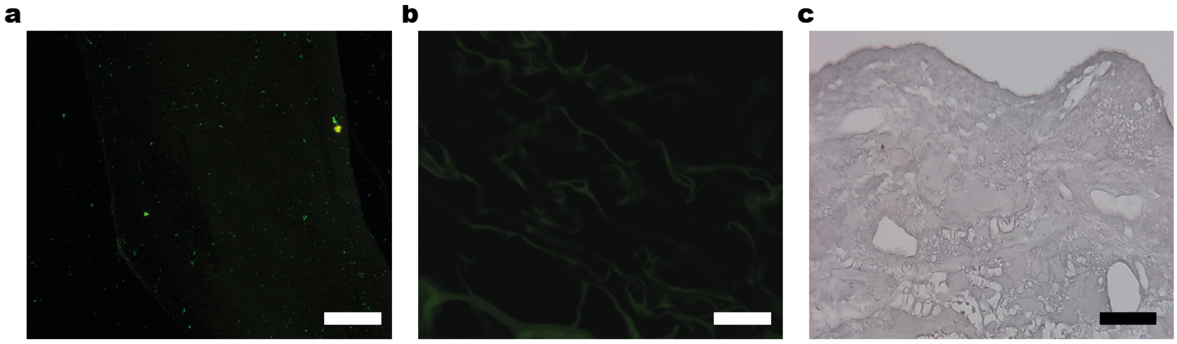

Supplementary Figure S1. Absence of dermatan sulfate in collagenous biomaterials before implantation/cell seeding. Immunostaining for dermatan sulfate (green) on **(a)** acellular bovine type I collagen hydrogel, **(b)** Integra® before implantation and **(c)** Glyaderm® before implantation. Note lack of staining. Scale bars are 50 μm.

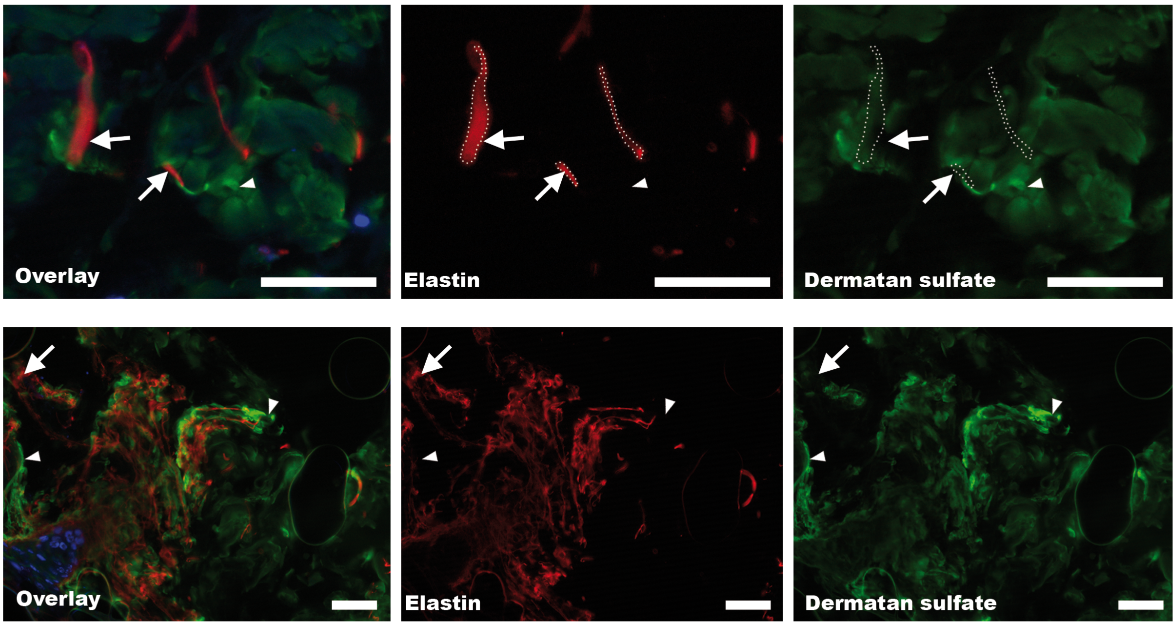

Supplementary Figure S2. Absence of dermatan sulfate on elastic fibers shown by double immunostaining of cryosections of sheep skin for elastin (red) and dermatan sulfate (green). Elastic fibers (red, arrow) are negative for dermatan sulfate (green, arrow head), and dermatan sulfate is negative for elastin staining. Scale bars are 50 μm.
